# Supplementary material for: Effect of emancipative values on life satisfaction across different levels of democracy: A cross-national analysis of the World Values Survey
Source: PLoS One. 2025 Jun 9;20(6):e0325198. doi: 10.1371/journal.pone.0325198 (PMC12148189; doi:10.1371/journal.pone.0325198)
Supplement: S1 Fig — (DOCX) [file pone.0325198.s001.docx]

**S1 Fig.** **Effect of emancipative values on life satisfaction at different levels of liberal democracy and historical contexts (2-10 years prior to survey)**


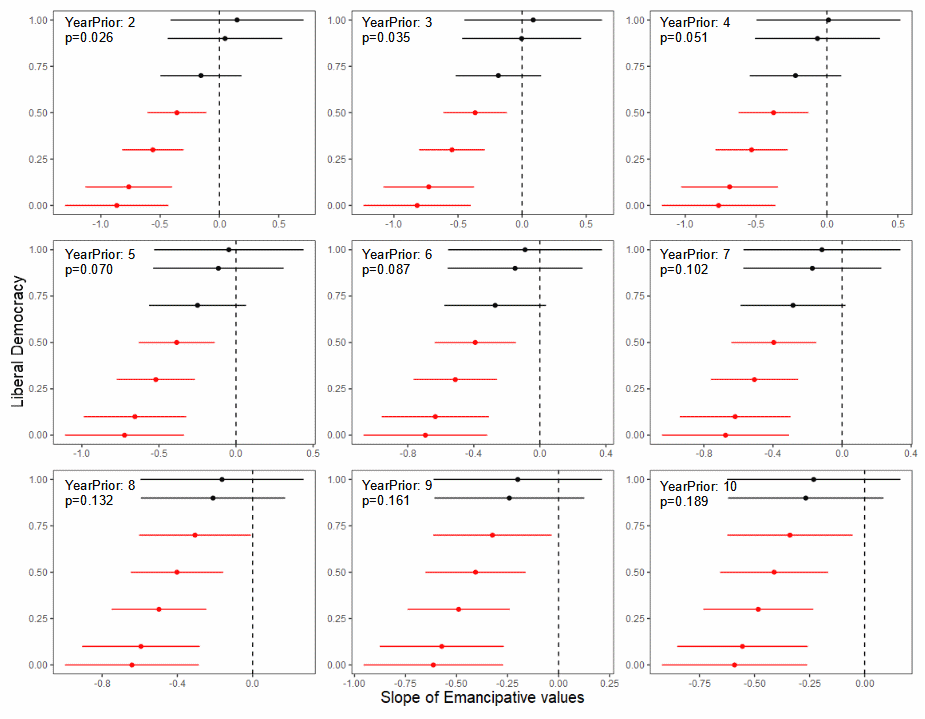


Effect of emancipative values on life satisfaction at varying liberal democracy indices, based on models incorporating different time lags for democracy measures prior to the survey year (2-10 years).
