# Supplementary material for: Effect of emancipative values on life satisfaction across different levels of democracy: A cross-national analysis of the World Values Survey
Source: PLoS One. 2025 Jun 9;20(6):e0325198. doi: 10.1371/journal.pone.0325198 (PMC12148189; doi:10.1371/journal.pone.0325198)
Supplement: Attachment — Submitted filename: Response to reviewers PloSOne.docx [file pone.0325198.s003.docx]

*Reviewer #1: The study investigates the relationship between emancipative values, operationalized as values that advocate for personal freedoms, equality, and autonomy, and life satisfaction across different levels of democracy. Using data from the seventh wave of the World Values Survey (WVS) and the Varieties of Democracy (V-Dem) dataset, the authors analyze how the impact of emancipative values on life satisfaction varies depending on the democratic context of a country. The study employs a random effects model to account for country-level variations and adjusts for individual-level factors such as income, education, and settlement size, as well as country-level factors like lagged income per capita and regional affiliation. The findings reveal a negative main effect of emancipative values on life satisfaction, but a significant positive interaction between democracy and emancipative values, suggesting that the effect of these values is context-dependent. In less democratic countries, emancipative values have a negative impact on life satisfaction, while in more democratic contexts, no significant effect is observed. The results challenge the conventional assumption that emancipative values universally enhance life satisfaction, highlighting the importance of cultural and institutional congruence, and suggesting that the alignment between individual values and societal norms plays a crucial role in determining well-being. The study also notes that the relationship between emancipative values and life satisfaction is more responsive to recent changes in democratic quality rather than long-term democratic traditions.*

**Response. Thank you for your positive feedback and thoughtful comments. Please find our responses to your points below.**

*he paper is rather short, with a very limited theoretical discussion. They refer to very limited availability of connected research. While one can concur with this fact, still a more dexterous work on the theoretical framework is missing, particularly with how the notion of democracy is presented. First, the paper makes no substantive mention to any of the relevant Political Science literature that could serve to contextualize how democracy is presented, beyond the concepts implied in the data selection. But authors have no advantage in saving the reader from such a discussion, leaving unanswered the important issue of a potential tautological issue, as the notion of emancipative values may be embedded in the very notion of democracy that they are using, which could explain the results in less developed countries. A more thorough discussion of what democracy is in this context is necessary, at least to remove this issue.*

**Response. We have expanded our theoretical framework in the Introduction (5^th^ paragraph, p. 4):**

At this point, it is important to recognize that while emancipative values are closely linked to democracy, they emerge at different levels: emancipative values arise at the individual level, whereas democracy operates at the level of governance or the state. In this sense, democracy provides the framework or enabling environment in which emancipative values can be expressed and realized. However, individuals living in democratic societies may not always hold emancipative values, and conversely, individuals in less democratic countries can still embrace and uphold emancipative values. Likewise, while there appears to be an obvious link between democracy and emancipative values, this connection is nuanced and influenced by several factors. To begin with, it is important to clarify what is meant by democracy, as it exists at different levels that may relate to emancipative values in varying degrees. As Coppedge et al. note, there is no consensus on a single definition of democracy beyond the basic idea of “rule by the people” (Coppedge 2022). In practice, most existing democracies are to some degree liberal and electoral, characterized by the rule of law, civil liberties, judicial independence, and free and fair elections of representatives. As conceptualized by the V-Dem project, there are three other key dimensions of democracy: participatory, deliberative, and egalitarian democracy, each capturing distinct but interconnected aspects of democratic life (Coppedge 2022).

Recent research highlights that emancipative values drive a “critical-liberal” demand for democracy, where individuals with strong emancipative values insist on democratic freedoms and hold high standards for democratic quality, including making rulers truly responsive to citizens’ rights and freedoms (Welzel et al. 2022), Likewise, electoral democracy serves as the essential mechanism that institutionalizes this responsiveness. Emancipative values motivate people to insist on genuine electoral competition, political pluralism, and the rejection of authoritarian or fraudulent practices (Welzel et al. 2022).

Other forms of democracy – deliberative, participatory, and egalitarian – also connect deeply with emancipative values by emphasizing broader citizen engagement, inclusive decision-making, and equality. For instance, John Dewey viewed democracy as a moral and social association in which individuals realize their full potential through active involvement in their communities and institutions (Caspary 2000). Dewey believed that such participation and deliberation are fundamental to individual freedom, extending beyond government into all areas of social life, fostering collaborative problem-solving and critical thinking.

Among the five dimensions of democracy, liberal democracy stands out as the most relevant domain for exploring emancipative values. As mentioned above, emancipation (autonomy, equality, choice, and voice) emphasizes values that align closely with liberal democracy (protection of rights, rule of law, and freedom to pursue one’s true self). Moreover, liberal democracy is perhaps the most tangible and visible form of democracy in our contemporary world, currently being seen as under threat, or alternatively as too hegemonic (Wolff 2023).”

*Regarding methodology, the explanatory power of the selected variables/ data is rather limited, as it is the linear analysis chosen. The authors acknowledge several limitations, saving the reader from the error of assuming automatic inferences. However, they state those limitations regarding several aspects, none of which related to very limited nature of linear analysis. For example, understood as systems, non-linear approaches may be more useful and less prone to frequentist approaches. Authors need to either more explicitly acknowledge this limitations in connection with the linear model selected, or enrich the analysis with eg non-linear, Bayesian or qualitative analysis.*

**Response. We have acknowledged the limitation inherent to the linearity of our analysis (Discussion, p. 15):**

Fifth, our random effects model assumed linearity and may not have captured potential non-linear dynamics or interactions. For example, the relationship between emancipative values and life satisfaction might follow a non-linear pattern (e.g., a bell curve) rather than a straightforward linear relationship. Interactions between emancipative values and factors such as age, gender, or income level could further nuance these relationships but were not explored here. Consequently, our findings should be interpreted as preliminary, highlighting the need for future studies to incorporate non-linear modeling and interaction effects.

*In general, this is a very brief but well written and sufficiently crafted paper, although at its current stage more appropriate as a preliminary analysis report, than as a fully fledged paper. Hower, as long as author/s extend the theoretical analysis as indicated, remarkably to more clearly discern the relation between democracy and the studied values; and as long as it more explicitly states its statsitical limitations or improve its explanatory power, it may be considered for publication.*

*Reviewer #2: This article answers a broad question about values, society, and life satisfaction using two strong and complementary datasets, one country-level expert-coded dataset about democratic institutions/norms and another individual-level dataset about individuals' values and attitudes across the globe. The authors analyze this dataset thoughtfully and carefully, such as using multilevel analysis to avoid committing an ecological fallacy (that mixes levels in less rigorous ways).

The strength of the research is that it tests a simple proposition from a prior theory about emancipative values enhancing life satisfaction, so long as basic needs have been met. They find contrary evidence, in two respects. In democracies, the values have no bearing on satisfaction. In less democratic countries, they have a negative association, perhaps owing to being frustrated by the undemocratic political (and social? economic?) system in which they live. Their main takeaway is to stress that one's context matters when considering what beliefs lead to a satisfying life. It's a straightforward finding, which makes abundant sense, and it provides an interesting anomaly for the aforementioned theory to consider. The weaknesses of the article seem relatively minor to me, in that each can be addressed by the authors with little difficulty.*

**Response. Thank you for your positive feedback and thoughtful comments. Please find our responses to your points below.** *First, I found the theoretical setup too sketchy, even for what is meant to be a brief article. Did the authors truly have no hypothesis? Page 4 provides the closest account of the authors' purpose, which is simply to "investigate" this question. A lack of predictive directional hypothesis makes the main finding seem like the catch pulled out of a fishing expedition. If the authors had a clearer vision, they should state it here. If not, then they could add to the Limitations section that they had non-directional hypotheses and can present only post-hoc directional findings.*

**Response. We clarify our hypotheses in the last paragraph of the Introduction (p. 5):**

We predicted that the democratic context would play a key role in shaping the effect of emancipative values on life satisfaction, manifesting as an interaction between liberal democracy and these values. The rising expectations model of revolution offers valuable insights to this hypothesis (Chandra and Foster 2005). In essence, the model suggests that citizens become frustrated when there is a gap between their heightened expectations for a better life and their actual experiences (with increasing gaps potentially sparking revolutionary unrest). Applied to our research, this implies that in countries with low levels of democracy, individuals with high emancipative values may experience lower life satisfaction due to these unmet expectations. In turn, increasing levels of democracy might not necessarily lead to higher life satisfaction among those with the highest emancipative values, as they may still feel discontented that their ideals remain unfulfilled.

*In either case, I'd like to see more theoretically grounded interpretation of these results. Surely they can find a stronger foundation or this pattern of outcomes. The "rising expectations" model of revolution came to mind, whereby people become frustrated by a govt as their expectations (for life, liberty, etc) rise--sparking revolution when the gap is too great. The authors don't say much about what these findings mean, nor how they fit into preexisting sociological work, political science research, and other scholarship.*

**Response. We have expanded our interpretation of the results ...**

**… At the end of the first paragraph of the Discussion (p. 12):**

When interpreting our findings, it is important to remember that our statistical estimates were derived from a model that controlled for other key predictors of life satisfaction (i.e. “all other things being equal”). In particular, we must emphasize that ensuring basic survival remains a fundamental concern, and that life satisfaction depends on far more than just the interaction between emancipative values and democracy. Nonetheless, the effect size for the interaction betweven emancipative values and the level of democracy approached a moderate magnitude and was, in absolute terms, larger than other statistically significant predictors of life satisfaction, such as income level, food security, and involvement in active memberships.

**… In the fourth paragraph, when discussing the effect in non-democracies (p. 13):**

Therefore, our results fit well with the rising expectations model of revolution which we briefly presented in the Introduction. Related to the model is the concept of relative deprivation, i.e. the discrepancy in what people expect to achieve and what they actually achieve (Abeles, 1976). According to this concept, it is this discrepancy, rather than absolute deprivation itself, that gives rise to dissatisfaction.

**… In the fifth paragraph, when discussing the effect as democracy increases (p. 13):**

Another hypothesis to explain this negative finding is that increasing freedoms raise expectations, and greater awareness of remaining restrictions or repression can feel intolerable, and in turn would not significantly alleviate dissatisfaction. This dynamic may occur because of what has been conceptualized as the “Tocqueville effect” (Elster 2009). When freedom and emancipation become dominant ideals in democratic societies, any visible repression clashes sharply with those ideals, fueling discontent. In addition, Tocqueville warns that although formal liberties may exist, the growth of centralized power and bureaucratic control can subtly undermine genuine freedom, leading to a different kind of dissatisfaction, here not merely because rising expectations are unmet, but because the exercise of freedom is constrained even as its appearance remains (Tocqueville 2002).

*A related suggestion is that the authors could provide better guidance for future research if they make clearer the theoretical stakes. I agree that scholars need to "explore the mechanisms" that could be at play in the results they find. But "exploration," per se, would repeat a weakness in here; that is, an exploratory approach suggests weak theoretical moorings for such future studies. Instead, I encourage the authors to posit potential rival mechanisms and how those might be tested. In a lab? In field experiments? In a longitudinal survey? If well conceived and described, this section of the article could be the key to inspiring well-designed future research that answers these questions more decisively.*

**Response. We have added a brief discussion of future research directions in the Conclusion (p. 15):**

Several potential mechanisms may underlie our findings. For instance, a diminished sense of belonging could result from a mismatch between a country’s level of democracy and an individual’s emancipative values. Other important mechanisms to consider include feelings of unfairness, injustice, and national pride. To better understand these competing explanations, we recommend collecting longitudinal survey data that track emancipative values and potential mediators over time, alongside life satisfaction as the outcome. This would enable the application of causal mediation analysis to more rigorously test these pathways.

*A more speculative comment comes from looking at the data. I'd like the authors to speak more vividly about effect size. How do the effects they see compare to related statistical associations? Are these tiny, small, or larger coefficients? And so on.*

**Response. We have included effects sizes in our Results section (p. 10):**

Our random effect model retrieved an overall negative effect of emancipative values on life satisfaction (b=-0.88; p<0.001) but no significant effect of liberal democracy (b=-0.37; p=0.41). However, there was a significant positive interaction between democracy and emancipative values (b=1.04; p=0.024). Standardized mean difference (Cohen's d) for the interaction between liberal democracy and emancipative values was of 0.46. As a point of comparison, other variables were predictive of life satisfaction such as income level (b=0.17; p<0.001; d=0.07), having no food (b=0.29; p<0.001 ; d=0.13), being involved in group memberships (b=0.10; p<0.001; d=0.04), settlement size (b=0.09; p<0.001; d=0.04), or being divorced, separated or widowed (b=-0.34; p<0.001; d=-0.15).

**We have also included an interpretation of these effect sizes (Discussion, p. 12):**

When interpreting our findings, it is important to remember that our statistical estimates were derived from a model that controlled for other key predictors of life satisfaction (i.e. “all other things being equal”). In particular, we must emphasize that ensuring basic survival remains a fundamental concern, and that life satisfaction depends on far more than just the interaction between emancipative values and democracy. Nonetheless, the effect size for the interaction between emancipative values and the level of democracy approached a moderate magnitude and was, in absolute terms, larger than other statistically significant predictors of life satisfaction, such as income level, food security, and involvement in active memberships.

*Looking at the figure, I also wonder if they could say more about their pattern of results. Currently, they write, "We found a significantly negative effect of emancipative values on 215 life satisfaction at low levels of democracy (liberal democracy index lower than 0.50), but no significant effect at 216 higher levels (Figure)." Fair enough, but researchers needn't only note what meets a p-value cutoff and what doesn't. (For that matter, some reviewers may be upset that the authors report as significant p values between .01 and .05, in spite of the enormous size of the dataset. That doesn't bother me, particularly for multilevel analysis, but there's dispute on this question.). What strikes me about the figure is that the mean effect size moves in a nearly linear fashion from least to most democratic societies. The way the authors describe it almost sounds like an s-shaped curve: suddenly associated below the .5 democracy cutoff, then just as suddenly non-sig above it. The pattern is more of a straight line. What does this mean? Could it be that as nations get *more* democratic, the positive association will, finally, become a significant one? The authors could note the democratic innovations literature and speculate out its payoff in the future.*

**Response. We briefly report such linear increase in our Result section (p. 10):**

There was a continuous increase in the mean estimate of emancipative values' effect on life satisfaction across societies ranging from least to most democratic. Moreover, we found a significantly negative effect of emancipative values on life satisfaction at low levels of democracy (liberal democracy index lower than 0.50), but no significant effect at higher levels (**Figure**).

**We also interpret this effect in the last paragraph of the Discussion (p. 14):**

Finally, this intricate relationship between democracy, emancipative values, and life satisfaction appears more pronounced when considering recent years of democracy rather than long-term trends. This suggests that the impact of emancipative values on life satisfaction is more responsive to recent changes in democratic quality rather than long-term democratic traditions. Looking ahead, what might happen as nations become even more democratic? One possibility is that the relationship between emancipative values and life satisfaction could eventually turn significantly positive, particularly as democratic innovations – such as citizen deliberation, direct democracy, or participatory governance – have demonstrated substantial societal benefits (He et al. 2022; Radcliff and Shufeldt 2016). Alternatively, following the “Tocqueville effect” (as previously discussed), individuals with heightened emancipative values might continually seek further progress, meaning the relationship between these values and life satisfaction could remain non-significant even in highly democratic societies. Another possibility is that if democracy reaches exceptionally high levels, individuals with lower emancipative values may experience strong dissatisfaction. Further research is needed to determine whether this dynamic will manifest in the future.
